# Supplementary material for: Abnormalities in the migration of neural precursor cells in familial bipolar disorder
Source: Dis Model Mech. 2022 Oct 18;15(10):dmm049526. doi: 10.1242/dmm.049526 (PMC9612872; doi:10.1242/dmm.049526)
Supplement: Supplementary information [file dmm-15-049526-s1.pdf]

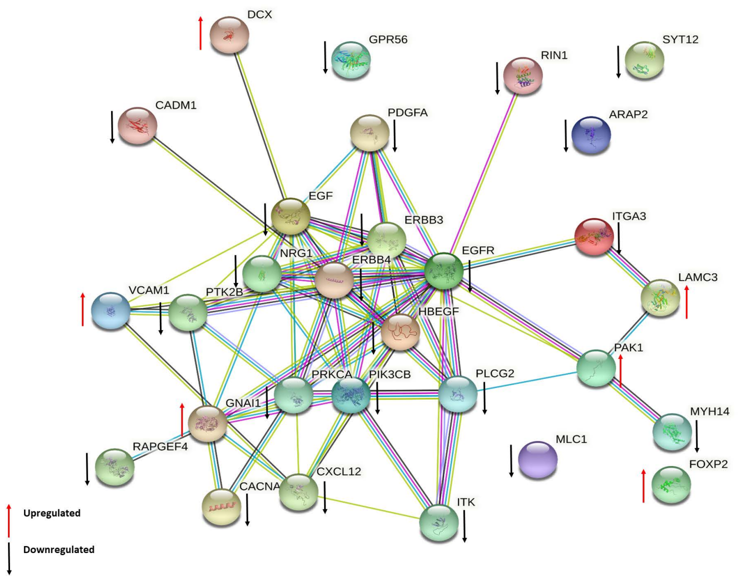

Fig. S1. Functional interactions among proteins shortlisted from Fig. 2C, explored using STRING online database. Only those proteins were included where direction of change of expression was similar in the two patient-derived lines.

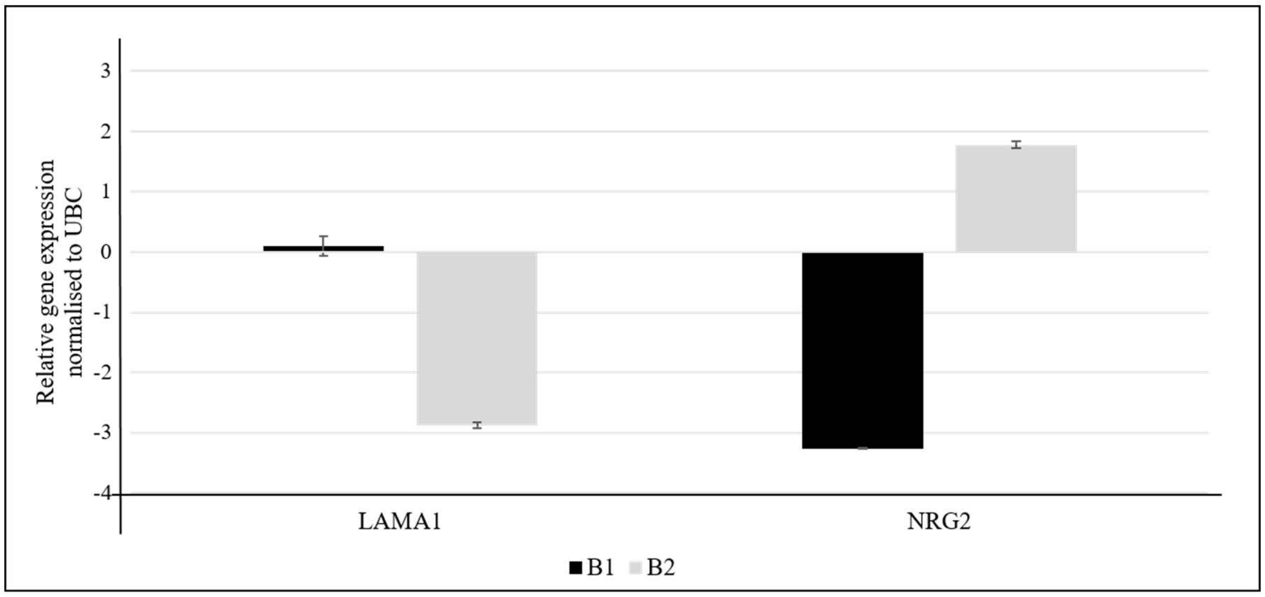

Fig. S2. Real-time qPCR analysis of *LAMA1* and *NRG2* gene expression in B1 and B2 lines. Data are represented as Mean ± SD from three samples per individual group of two independent experiments.

Disease Models & Mechanisms • Supplementary information

Table S1. Rare damaging exome variants identified in patients

Gene	Variant	RS id/Novel	No of affected	Presence in B1/B2	Signaling pathway	Cellular role
**DENND5A**	c.A2699G	rs779817963	4	B1/B2	ERK pathway (Han et al.,2016)	Cell migration, proliferation, apoptosis (Li et al., 2014)
**KIF7**	c.G2690C	rs749711306	3	B1	Hedgehog signalling. (He et al., 2014; Dafinger et al., 2011)	Cell proliferation, migration (Ho et al., 2014)
**SCN3A**	c.G83A	rs775711350	3	B1/B2	No pathway reported	Neuronal migration (Brackenbury et al., 2008), Cell cycle (Besson et al., 2015)
**PARP14**	c.G3467A	Novel	4	B1/B2	JNK2 signalling (Iansante et al., 2015)	Apoptosis (Barbarulo et al., 2013), glycolysis (Cho et al., 2011)
**PCCB**	c.C595T	rs371155999	3	B1/B2	Proprionate metabolism pathway (Porntaveetus et al., 2015)	Mitochondrial oxidative phosphorylation (Chapman et al., 2018)
**TRMT44**	c.C1405T	rs373816157	3	B1		
**NRG2**	c.C1477T	rs148371256	3	B1	MAPK and ERBB pathway (Benzel et al., 2007; Falls 2003)	Cell migration, proliferation (Ghashghaei et al., 2006; Wu et al., 2019), oxidative stress (Vyas et al., 2005)
**NIPBL**	c.A4496C	Novel	4	B1/B2	Notch pathway (Pistocchi et al., 2013), Cohesion (Liu et al., 2009), Wnt and PI3K-AKT pathway (Pistocchi et al., 2013)	Cell migration, proliferation, apoptosis (Xu et al., 2015; Yuen et al., 2016)
**SCUBE3**	c.C1996T	Novel	3	B1/B2	FGF Pathway (Tu et al., 2014) and hedgehog signaling (Xavier et al., 2013)	Cell proliferation (Liang et al., 2015)
**ANLN**	c.C128T	rs575071809	3	B1/B2	PI3K pathway.(Zeng et al., 2017)	Cell migration, Cell cycle (Magnusson et al., 2016; Zhou et al., 2015)

Disease Models & Mechanisms • Supplementary information

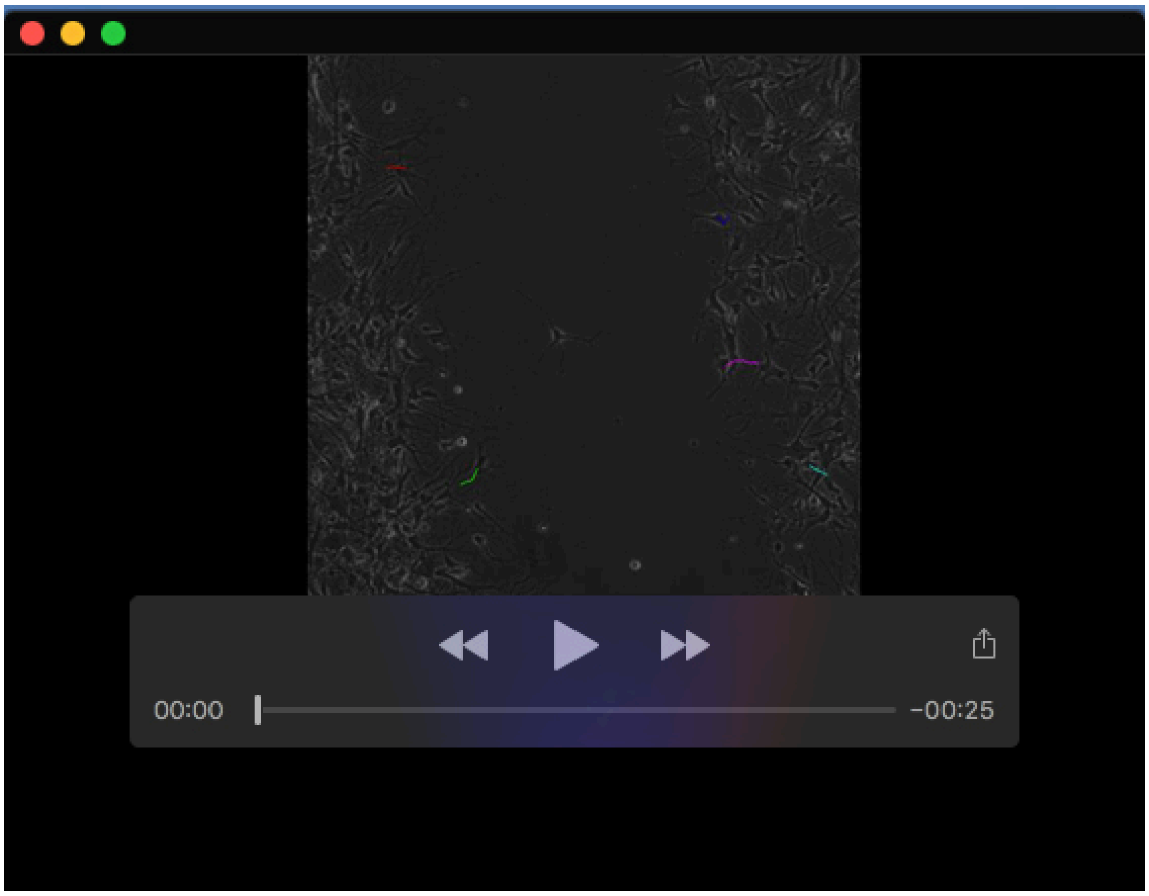

Movie 1. Representative recordings of cellular migration in C1 line. Migration tracks of individual cells were recorded by marking the position of the nucleus in individual frames. Colored lines indicate positions of individual cells during the duration of migration assay. The migration pattern in C1 is largely linear, showing individual cells from either side of 500 μm gap. Images for migration of C1 was captured with 4x objective.

Disease Models & Mechanisms • Supplementary information

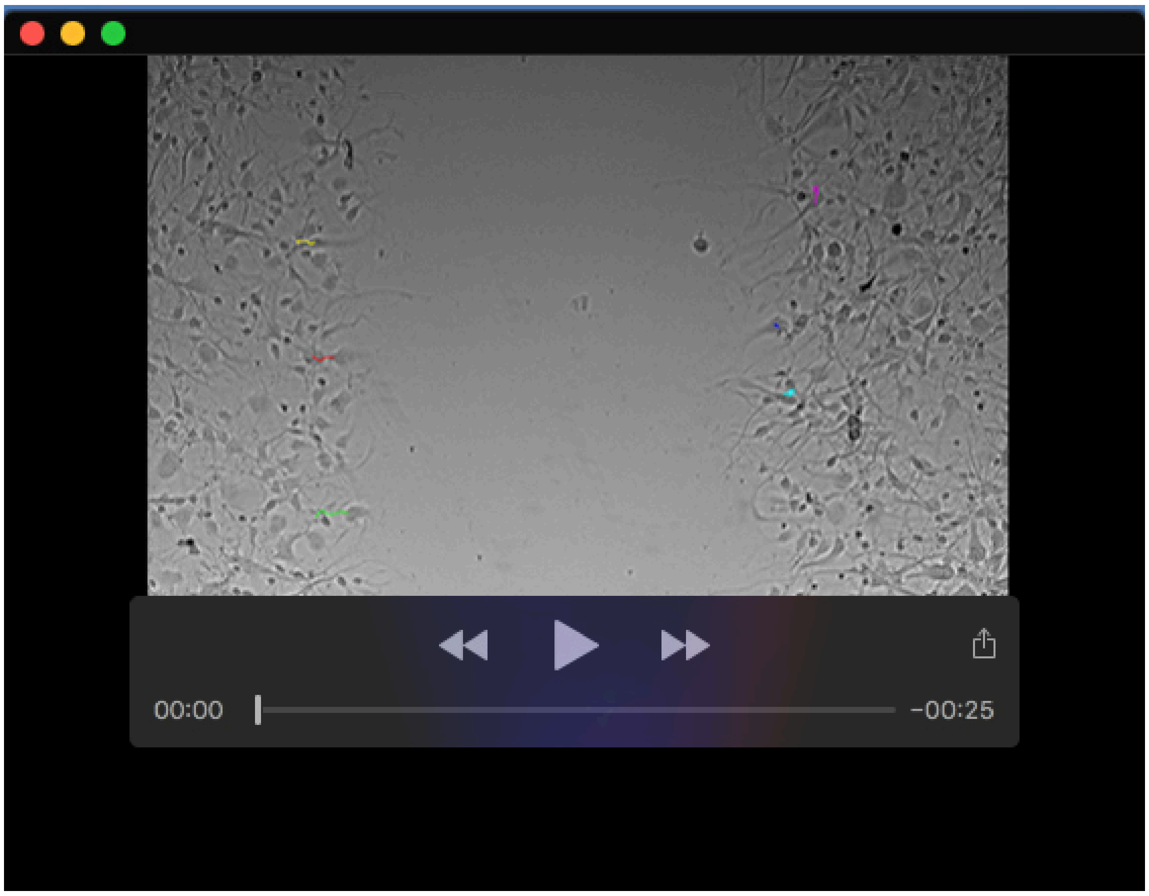

Movie 2. Representative recordings of cellular migration in C2 line. Migration tracks of individual cells were recorded by marking the position of the nucleus in individual frames. Colored lines indicate positions of individual cells during the duration of migration assay. The migration pattern in C2 is largely linear, showing individual cells from either side of 500 µm gap. Images for migration of C2 was captured with 10x objective.

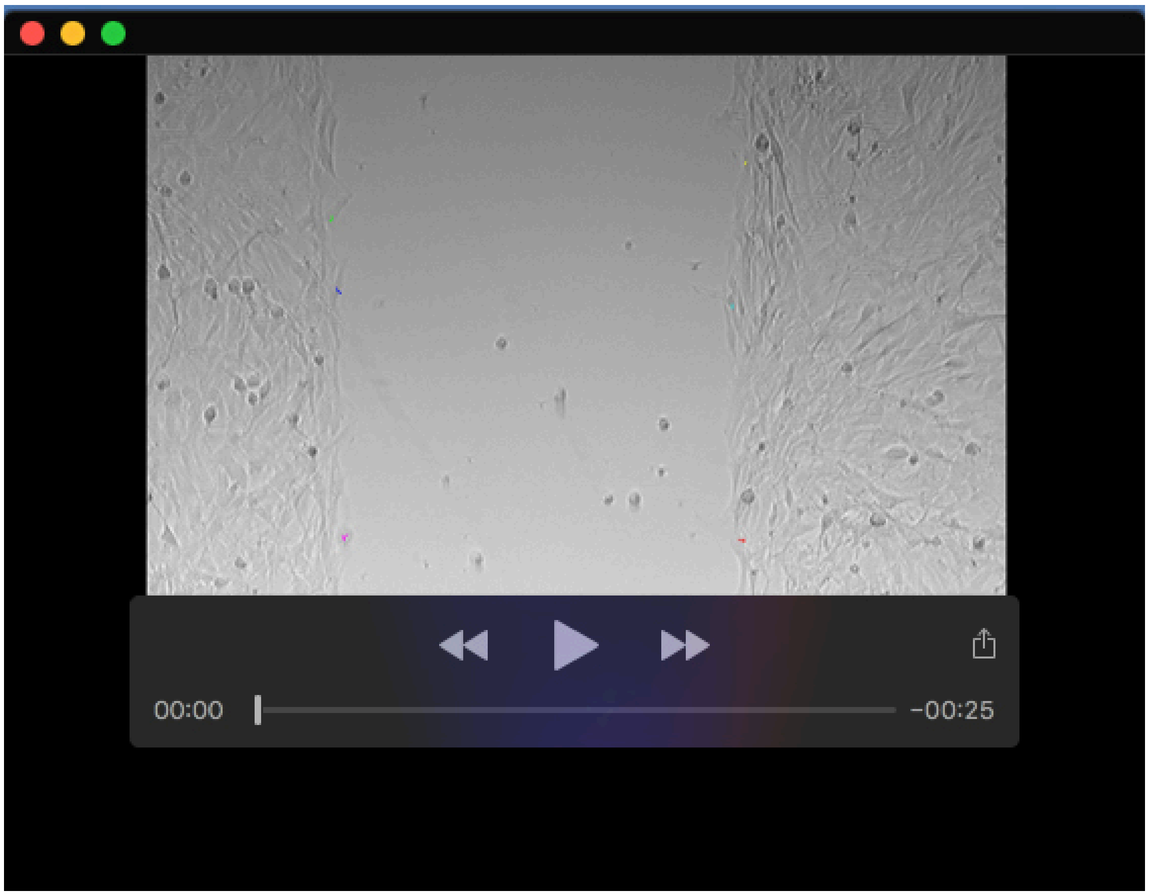

Movie 3. Representative recordings of cellular migration in C3 line. Migration tracks of individual cells were recorded by marking the position of the nucleus in individual frames. Colored lines indicate positions of individual cells during the duration of migration assay. The migration pattern in C3 is largely linear, showing individual cells from either side of 500 μm gap. Images for migration of C3 was captured with 10x objective.

Disease Models & Mechanisms • Supplementary information

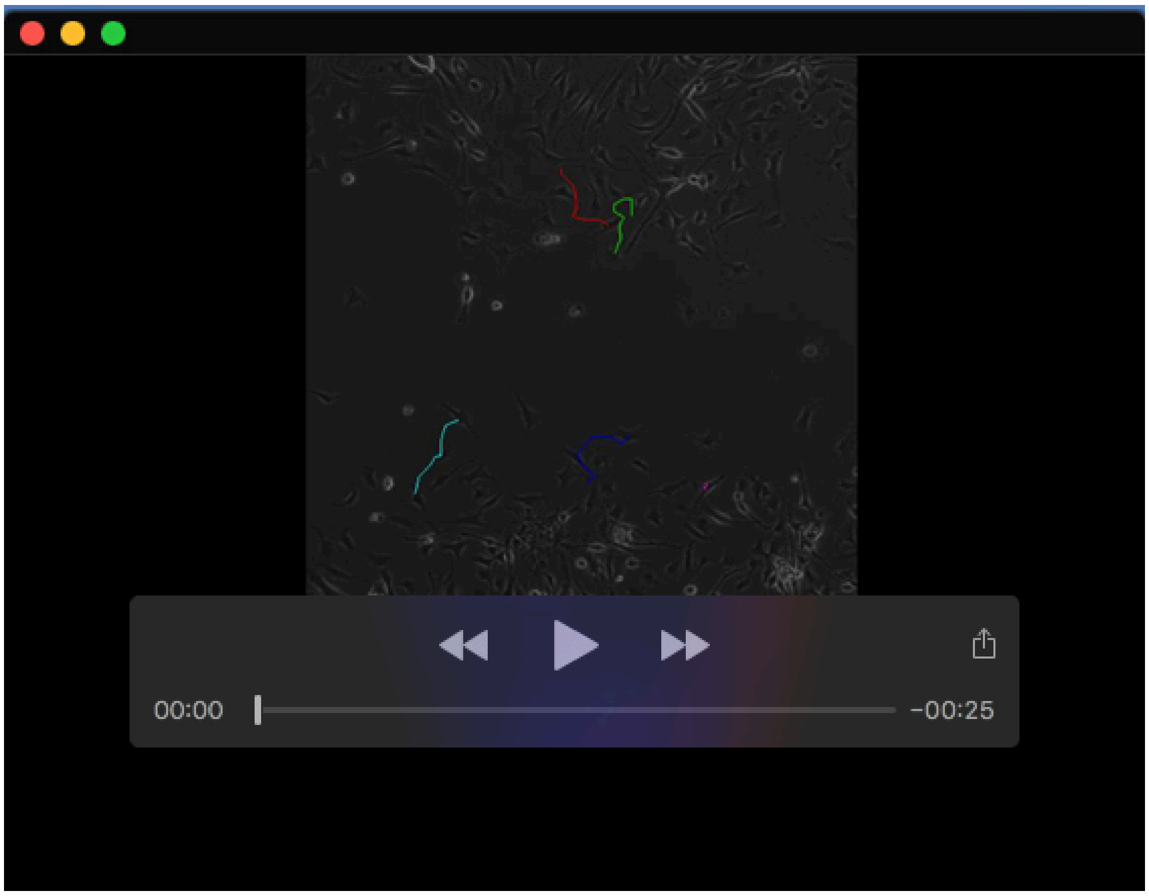

Movie 4. Representative recordings of cellular migration in B1 line. Migration tracks of individual cells were recorded by marking the position of the nucleus in individual frames. Colored lines indicate positions of individual cells during the duration of migration assay. From the migration pattern in B1, we encountered mixed patterns of cellular migration: apart from linear trajectory, these NPCs also demonstrated back-and-forth as well as circular motions. Images for migration of B1 was captured with 4x objective.

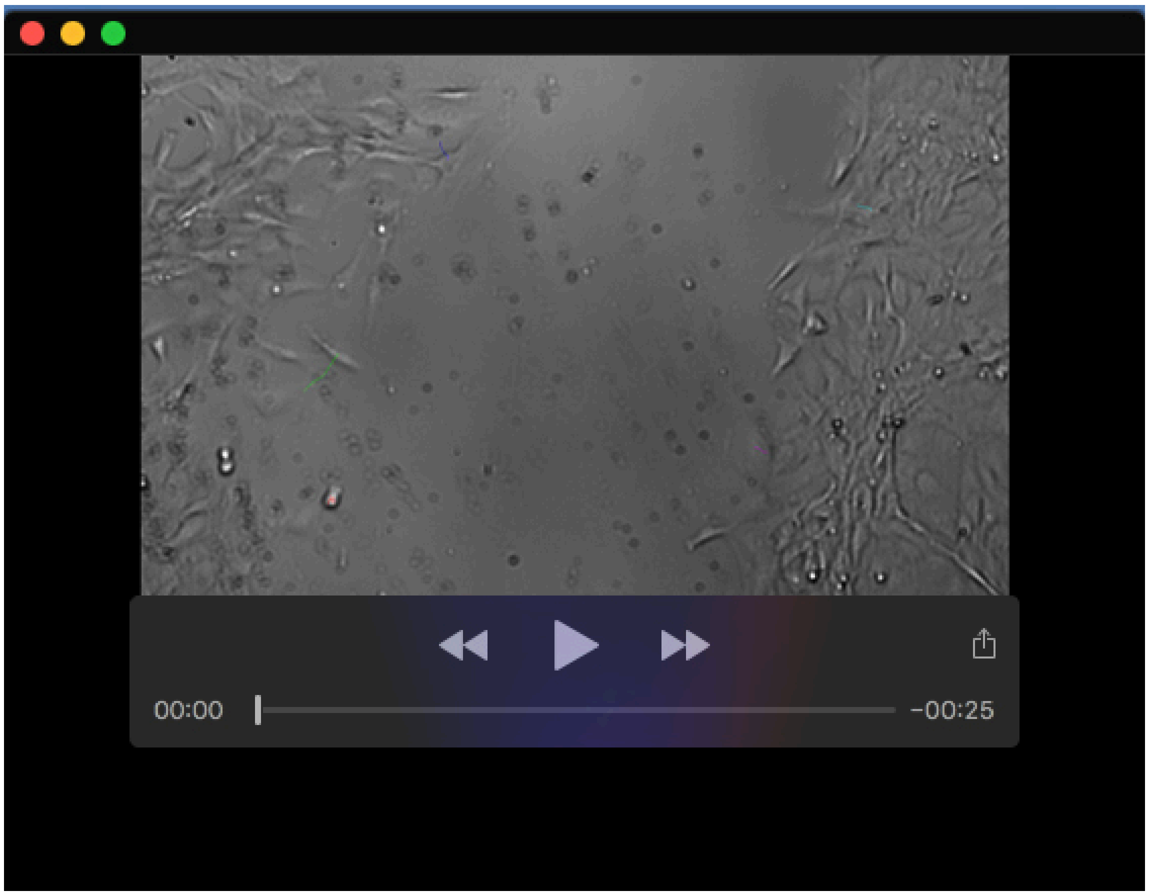

Movie 5. Representative recordings of cellular migration in B2 line. Migration tracks of individual cells were recorded by marking the position of the nucleus in individual frames. Colored lines indicate positions of individual cells during the duration of migration assay. From the migration pattern in B2, we encountered mixed patterns of cellular migration: apart from linear trajectory, these NPCs also demonstrated back-and-forth as well as circular motions. Images for migration of B2 was captured with 10x objective.

While the migration pattern in C1-C3 is largely linear (S1-A, B, C), showing individual cells from either side of 500µm gap; in B1 and B2 lines (S1-D and S1-E), we encountered mixed patterns of cellular migration: apart from linear trajectory, these NPCs also demonstrated back-and-forth as well as circular motions. Images for migration of C1 and B1 cells were captured with 4x objective and C2, C3, B2 cell migration captured with 10x objective.

References

Han, C., Alkhater, R., Froukh, T., Minassian, A.G., Galati, M., Liu, R.H., Fotouhi, M., Sommerfeld, J., Alfrook, A.J,, Marshall, C., et al. (2016). Epileptic Encephalopathy Caused by Mutations in the Guanine Nucleotide Exchange Factor DENND5A. *Am J Hum Genet.* **99**, 1359–1367.

Li, Y., Xu, J., Xiong, H., Ma, Z., Wang, Z., Kipreos, E.T., Dalton, S. and Zhao, S. (2014). Cancer driver candidate genes AVL9, DENND5A and NUPL1 contribute to MDCK cystogenesis. *Oncoscience*. **1**:854–865.

He, M., Subramanian, R., Bangs, F., Omelchenko, T., Liem, K.F., Kapoor, T.M. and Anderson, K.V. (2014). The kinesin-4 protein Kif7 regulates mammalian Hedgehog signalling by organizing the cilium tip compartment. *Nat Cell Biol*. **16**, 663–672.

Dafinger, C., Liebau, M.C., Elsayed, S.M., Hellenbroich, Y., Boltshauser, E., Korenke, G.C., Fabretti, F., Janecke, A.R,, Ebermann, I., Nürnberg, G. et al. (2011). Mutations in KIF7 link Joubert syndrome with Sonic Hedgehog signaling and microtubule dynamics. *J Clin Invest*. **121,** 2662–2667.

Ho, J., Du, Y., Wong, OG-W., Siu, M.K.Y., Chan, K.K.L. and Cheung, A.N.Y. (2014). Downregulation of the Gli Transcription Factors Regulator Kif7 Facilitates Cell Survival and Migration of Choriocarcinoma Cells. *PLoS One*. **9**:e108248.

Brackenbury, W.J., Djamgoz, M.B.A. and Isom, L.L. (2008). An Emerging Role for Voltage-Gated Na+ Channels in Cellular Migration: Regulation of Central Nervous System Development and Potentiation of Invasive Cancers. *Neurosci.* **14,** 571–583.

Besson, P., Driffort, V., Bon, É., Gradek, F., Chevalier, S. and Roger, S. (2015). How do voltage-gated sodium channels enhance migration and invasiveness in cancer cells? *Biochim Biophys Acta - Biomembr*. **1848,** 2493–2501.

Iansante, V., Choy, P.M., Fung, S.W., Liu, Y., Chai, J.G., Dyson, J., Del, R.A., D'Santos, C., Williams, R., Chokshi, S., et al. (2015). PARP14 promotes the Warburg effect in hepatocellular carcinoma by inhibiting JNK1-dependent PKM2 phosphorylation and activation. *Nat Commun*. **6**:7882.

Barbarulo, A., Iansante, V., Chaidos, A., Naresh, K., Rahemtulla, A., Franzoso, G., Karadimitris, A., Haskard, D.O., Papa, S. and Bubici, C. (2013). Poly(ADP-ribose) polymerase family member 14 (PARP14) is a novel effector of the JNK2-dependent pro-survival signal in multiple myeloma. *Oncogene*. **32,** 4231–4242.

Cho, S.H., Ahn, A.K., Bhargava, P., Lee, C.H., Eischen, C.M., McGuinness, O. and Boothby, M. (2011). Glycolytic rate and lymphomagenesis depend on PARP14, an ADP ribosyltransferase of the B aggressive lymphoma (BAL) family. *Proc Natl Acad Sci U S A.***108,** 15972–15977.

Porntaveetus, T., Srichomthong, C., Suphapeetiporn, K. and Shotelersuk, V. (2015). A novel PCCB mutation in a Thai patient with propionic acidemia identified by exome sequencing. *Hum Genome Var*. **2**, 15033.

Chapman, K.A., Ostrovsky, J., Rao, M., Dingley, S.D., Polyak, E., Yudkoff, M., Xiao, R., Bennett, M.J. and Falk, M.J. (2018). Propionyl-CoA carboxylase pcca-1 and pccb-1 gene deletions in Caenorhabditis elegans globally impair mitochondrial energy metabolism. *J Inherit Metab Dis.* **41,** 157–168.

Disease Models & Mechanisms • Supplementary information

Benzel, I., Bansal, A., Browning, B.L., Galwey, N.W., Maycox, P.R., McGinnis, R., Smart, D., St Clair, D., Yates, P. and Purvis, I. (2007). Interactions among genes in the ErbB-Neuregulin signalling network are associated with increased susceptibility to schizophrenia. *Behav Brain Funct*. **3,** 31.

Falls, D.L. (2003). Neuregulins: functions, forms, and signaling strategies. *Exp Cell Res*. **284,** 14–30.

Ghashghaei, H.T., Weber, J., Pevny, L., Schmid, R., Schwab, M.H., Lloyd, K.C., Eisenstat, D.D., Lai, C. and Anton, E.S. (2006). The role of neuregulin-ErbB4 interactions on the proliferation and organization of cells in the subventricular zone. *Proc Natl Acad Sci U S A*. **103,** 1930–1935.

Wu, J., Li, M. and Zhang, Y. (2019). Long noncoding RNA *HOXA-AS2* regulates the expression of *SCN3A* by sponging *miR-106a* in breast cancer. *J Cell Biochem.* **120,** 14465–14475.

Vyas, V.K., Berkey, C.D., Miyao, T. and Carlson, M. (2005). Repressors Nrg1 and Nrg2 regulate a set of stress-responsive genes in Saccharomyces cerevisiae. *Eukaryot Cell*. **4**, 1882–1891.

Pistocchi, A., Fazio, G., Cereda, A., Ferrari, L., Bettini, L.R., Messina, G., Cotelli, F., Biondi, A., Selicorni, A. and Massa, V. (2013). Cornelia de Lange Syndrome: NIPBL haploinsufficiency downregulates canonical Wnt pathway in zebrafish embryos and patients fibroblasts. *Cell Death Dis*. **4,** e866.

Liu, J., Zhang, Z., Bando, M., Itoh, T., Deardorff, M.A., Clark, D., Kaur, M., Tandy, S., Kondoh, T., Rappaport, E., et al. (2009). Transcriptional Dysregulation in NIPBL and Cohesin Mutant Human Cells. *PLoS Biol*. **7,** e1000119.

Xu, W., Ying, Y., Shan, L., Feng, J., Zhang ,S., Gao, Y., Xu, X., Yao, Y., Zhu, C. and Mao, W. (2015). Enhanced expression of cohesin loading factor NIPBL confers poor prognosis and chemotherapy resistance in non-small cell lung cancer. *J Transl Med.* **13,** 153.

Yuen, K.C., Xu, B., Krantz, I.D. and Gerton, J.L. (2016). NIPBL Controls RNA Biogenesis to Prevent Activation of the Stress Kinase PKR. *Cell Rep*. **14**, 93–102.

Tu, C.F., Tsao, K.C., Lee, S.J. and Yang, R.B. (2014). SCUBE3 (Signal Peptide-CUB-EGF Domain-containing Protein 3) Modulates Fibroblast Growth Factor Signaling during Fast Muscle Development. *J Biol Chem.* **289,** 18928–18942.

Xavier, G.M., Panousopoulos, L. and Cobourne, M.T. (2013). Scube3 Is Expressed in Multiple Tissues during Development but Is Dispensable for Embryonic Survival in the Mouse. *PLoS One.* **8,** e55274.

Liang, W., Yang, C., Peng, J., Qian, Y. and Wang, Z. (2015). The Expression of HSPD1, SCUBE3, CXCL14 and Its Relations with the Prognosis in Osteosarcoma. *Cell Biochem Biophys.* **73,** 763–768.

Zeng, S., Yu, X., Ma, C., Song, R., Zhang, Z., Zi, X., Chen, X., Wang, Y., Yu, Y., Zhao, J., et al. (2017). Transcriptome sequencing identifies ANLN as a promising prognostic biomarker in bladder urothelial carcinoma. *Sci Rep*. **7,** 3151.

Magnusson, K., Gremel, G., Rydén, L., Pontén, V., Uhlén, M., Dimberg, A., Jirström, K. and Pontén, F. (2016). ANLN is a prognostic biomarker independent of Ki-67 and essential for cell cycle progression in primary breast cancer. *BMC Cancer*. **16,** 904.

Zhou, W., Wang, Z., Shen, N., Pi, W., Jiang, W., Huang, J., Hu, Y., Li, X., Sun, L. (2015). Knockdown of ANLN by lentivirus inhibits cell growth and migration in human breast cancer. *Mol Cell Biochem*. **398,** 11–19.
